# Xylan extraction from pretreated sugarcane bagasse using alkaline and enzymatic approaches

**DOI:** 10.1186/s13068-017-0981-z

**Published:** 2017-12-07

**Authors:** Daniele Sporck, Felipe A. M. Reinoso, Jorge Rencoret, Ana Gutiérrez, José C. del Rio, André Ferraz, Adriane M. F. Milagres

**Affiliations:** 1 Departamento de Biotecnologia, Escola de Engenharia de Lorena, Universidade de São Paulo, Lorena, SP 12602-810 Brazil; 2 Instituto de Recursos Naturales y Agrobiología de Sevilla, CSIC, Av. Reina Mercedes, 10, 41012 Seville, Spain

**Keywords:** Xylan, Sugarcane, Biorefinery, Cellulose hydrolysis, Alkaline-sulfite pretreatment

## Abstract

**Background:**

New biorefinery concepts are necessary to drive industrial use of lignocellulose biomass components. Xylan recovery before enzymatic hydrolysis of the glucan component is a way to add value to the hemicellulose fraction, which can be used in papermaking, pharmaceutical, and food industries. Hemicellulose removal can also facilitate subsequent cellulolytic glucan hydrolysis.

**Results:**

Sugarcane bagasse was pretreated with an alkaline-sulfite chemithermomechanical process to facilitate subsequent extraction of xylan by enzymatic or alkaline procedures. Alkaline extraction methods yielded 53% (w/w) xylan recovery. The enzymatic approach provided a limited yield of 22% (w/w) but produced the xylan with the lowest contamination with lignin and glucan components. All extracted xylans presented arabinosyl side groups and absence of acetylation. 2D-NMR data suggested the presence of *O*-methyl-glucuronic acid and *p*-coumarates only in enzymatically extracted xylan. Xylans isolated using the enzymatic approach resulted in products with molecular weights (Mw) lower than 6 kDa. Higher Mw values were detected in the alkali-isolated xylans. Alkaline extraction of xylan provided a glucan-enriched solid readily hydrolysable with low cellulase loads, generating hydrolysates with a high glucose/xylose ratio.

**Conclusions:**

Hemicellulose removal before enzymatic hydrolysis of the cellulosic fraction proved to be an efficient manner to add value to sugarcane bagasse biorefining. Xylans with varied yield, purity, and structure can be obtained according to the extraction method. Enzymatic extraction procedures produce high-purity xylans at low yield, whereas alkaline extraction methods provided higher xylan yields with more lignin and glucan contamination. When xylan extraction is performed with alkaline methods, the residual glucan-enriched solid seems suitable for glucose production employing low cellulase loadings.

**Electronic supplementary material:**

The online version of this article (10.1186/s13068-017-0981-z) contains supplementary material, which is available to authorized users.

## Background

Sugarcane bagasse is a valuable lignocellulosic material to produce fuels and chemicals because it is site-available at the current sugar and ethanol mills [1]. However, advanced chemical and material applications of sugarcane bagasse components require suitable pretreatment technologies that facilitate efficient valorization of cellulose, hemicellulose, and lignin fractions. Several pretreatment methods have been developed to open the compact lignocellulose ultrastructure and make separations or conversions easier [2, 3].

The alkaline-sulfite chemithermomechanical treatment is considered suitable for this purpose because it is a delignification process that retains most of the cellulose and a significant fraction of hemicellulose in the pretreated solids. In addition, lignocellulose pretreatment with sulfite ions makes residual lignin sulfonated and hydrophilic, increasing the swelling and porosity of the cell walls [4, 5]. By properly controlling reaction time, temperature, and pH, it is possible to decrease the lignocellulose recalcitrance through a mild sulfite pretreatment process [4, 6–10].

In previous studies, efficient sugar production from alkaline-sulfite-pretreated sugarcane bagasse was obtained by simultaneous enzymatic hydrolysis of cellulose and hemicelluloses [5, 9, 11]. However, yeasts currently used in industrial fermentation processes do not efficiently ferment the blend of hexoses and pentoses, resulting in carbohydrate losses in the final product [12, 13].

Considering the characteristics of the alkaline-sulfite pretreatment, we envisage that the residual hemicellulose is susceptible to be isolated in its polymeric form with commercial applicability. Current examples of hemicellulose uses include packaging films, coating food products, biomedical products, and additives for cellulose fibers [14–19].

Hemicelluloses are the second most abundant renewable material produced by plants, and xylan is the major hemicellulose present in sugarcane bagasse secondary cell walls. Xylan is composed of an anhydrous xylose backbone with a high degree of substitution, which has a direct effect on its properties [20–23]. The impact of hemicelluloses properties, such as molecular weight and degree of substitution for different applications, is widely discussed in the literature. Research has shown that hemicelluloses with a high degree of uronic acid or other side chains are not easily adsorbed onto pulp fibers [24–26]. However, which molecular weight range of the hemicelluloses is more effective for adsorption on pulp fibers remains in question. For example, Magaton et al. [27] reported that higher molecular weight hemicelluloses are more effective than those with lower molecular weight, while Muguet et al. [16] found that smaller molecules tend to form large aggregates and thus deposit more efficiently onto fibers. A more specific backbone length of at least 15 unsubstituted xylosyl residues was determined for xylan adsorption on bacterial cellulose [28].

Methods for xylan extraction are chosen based on compromises among extraction yield, selectivity, and polymerization degree of xylans [18]. Polymeric xylans suitable for biopolymer applications are generally pre-extracted from raw lignocellulosic materials in alkaline solutions; however, lignin fractions are also solubilized [29]. Recently, Chimphango et al. [23] and Carvalho et al. [30] tested specific extraction procedures from untreated sugarcane bagasse. Traditional hemicellulose extraction methods include lignocellulose pre-delignification with sodium chlorite followed by alkaline extraction, which provides high yields of polymeric xylan [31]. A second traditional method is based on direct alkaline extraction followed by ethanol precipitation of xylan, which is a simpler procedure but usually provides a lower yield [32]. Both methods use 40% (w/w) NaOH for xylan extraction at different reaction times and temperatures. The use of these methods for direct extraction of xylan from sugarcane bagasse indicated a higher yield when bagasse was pre-delignified [23]. Even so, the purities of the isolated xylans were low, since 16–28% of residual lignin was detected in the extracted xylans [23]. Other studies combine enzymatic treatments with alkaline extraction to remove xylan and xylo-oligosaccharides from lignocellulosic materials [33–35]. In these studies, high enzyme loads resulted in the extraction of xylans with low molecular weight. Aguedo et al. [35] used xylanases for enzymatic extraction of arabinoxylans from wheat bran, reaching low yields (8%) of a high-molecular-weight product (5–12.5 kDa). Remond et al. [33] studied the effect of wheat straw pretreatment with ammonia and subsequent endo-xylanase (purified from *Thermobacillus xylanilyticus*) removal of xylan. The authors found that the maximum xylan solubilization was 57% when the process used 350 IU xylanase per gram of pulp.

The current work used alkaline-sulfite-pretreated sugarcane bagasse as a feedstock for xylan extraction instead of directly recovering the polymer from untreated lignocellulose. Here, we compare two traditional alkaline extraction procedures with an enzymatic extraction method performed at mild alkaline conditions. The goal is to use the enzymatic approach to prepare the purest xylans under mild alkaline extraction conditions. All process steps were designed in a biorefinery concept where xylan is recovered before enzymatic hydrolysis of the glucan component, which can add value to the hemicellulose fraction, and provides a pentose-free hydrolysate suitable for conventional industrially developed fermentation processes.

## Methods

### Materials

Beechwood xylan was obtained from Megazyme (lot 141101a) and sugarcane bagasse was supplied by a sugar and ethanol mill located in São Paulo state, Brazil. The analytical grade sugars glucose, cellobiose, arabinose, xylose, and glucuronic acid were obtained from Sigma-Aldrich at purity levels above 99% and were used as standards in sugar analysis. Commercial xylanase (Luminase-PB 200, BASF) was used for enzymatic extraction of xylan. Commercial cellulases were used in hydrolysis experiments (cellulases from *Trichoderma reesei* ATCC 26921 and Novozym 188 β-glucosidase, both purchased from Sigma-Aldrich). Sugarcane bagasse was provided by a sugar and ethanol mill located at the São Paulo state, Brazil, which uses a mix of commercial hybrids of sugarcane cultivars. The sample was obtained just after crushing for juice extraction. Resulting bagasse was air-dried and stored with approximately 12% humidity.

### Alkaline-sulfite chemithermomechanical pretreatment of sugar cane bagasse

Sugarcane bagasse was submitted to alkaline-sulfite pretreatment as described by Mendes et al. [5], avoiding the step of extensive washing of the pretreated material, which was named unwashed solids. The alkaline-sulfite load used in the pretreatment was set to 5% NaOH and 10% Na_2_SO_3_, both expressed as gram of reagent per 100 g of oven-dry sugarcane bagasse.

### Hemicellulose extraction

The hemicellulose fractions were extracted from alkaline-sulfite-pretreated sugarcane bagasse using two previously described methods [31, 32]. Both methods were based on alkaline hemicellulose extraction, being previously evaluated for direct xylan extraction from untreated sugarcane bagasse [23]. In summary, the Lopez method was based on GAX extraction with 40% NaOH (w/w) at 60 °C for 2 h [32], while the Hoije method uses the same alkaline charge but a lower temperature (25 °C) and a longer extraction period (16 h). The Hoije method also differs from the Lopez method by using a previous acid-chlorite delignification [31]. Enzymatic extraction of hemicellulose was performed by treating the alkaline-sulfite-pretreated sugarcane bagasse with commercial xylanase with a dosage of 8 IU/g of pretreated bagasse at 5% consistency in 50 mM phosphate buffer at pH 8 and 50 °C. During the enzymatic treatment, the samples were mixed at 120 rpm for 24 h. After the treatment, the material was heated to 90 °C for 15 min to inactivate the enzymes, the residual solids were removed by filtration, and xylans were recovered after freeze-drying of the soluble fraction [34].

### Chemical characterization of the samples

Untreated and pretreated sugarcane bagasse were extracted with 95% ethanol for 6 h in a Soxhlet apparatus before characterization. All samples were characterized based on acid hydrolysis procedures previously published [36]. Concentrations of monomeric sugars and acetic acid were determined by HPLC using a BIO-RAD HPX87H column at 45 °C eluted at 0.6 mL/min with 5 mM sulfuric acid detected with a temperature-controlled RI detector. The uronic acid contents of beechwood xylan and sugarcane-extracted xylans were determined using the carbazole–sulfuric acid method, as previously described by Brienzo et al. [22].

2D-NMR characterization of the samples used approximately 50 mg of xylan. Samples were dissolved in 0.75 mL of DMSO-*d*_6_. NMR spectra were recorded at 25 °C on a Bruker AVANCE III 500 MHz instrument equipped with a cryogenically cooled 5 mm TCI gradient probe with inverse geometry (proton coils closest to the sample). HSQC (heteronuclear single quantum coherence) experiments used Bruker’s ‘hsqcetgpsisp2.2’ pulse program (adiabatic-pulsed version) with spectral widths of 5000 Hz (from 10 to 0 ppm) and 23,843 Hz (from 165 to 0 ppm) for the ^1^H- and ^13^C-dimensions. The number of collected complex points was 2048 for the ^1^H-dimension, with a recycle delay of 1 s. The number of transients was 64, and 256 time increments were always recorded in the ^13^C-dimension. The ^1^ *J*_CH_ used was 145 Hz. Processing used typical matched Gaussian apodization in the ^1^H dimension and squared cosine-bell apodization in the ^13^C dimension. Prior to Fourier transformation, the data matrixes were zero-filled up to 1024 points in the ^13^C-dimension. The central solvent peak was used as an internal reference (*δ*_C_ 39.5; *δ*_H_ 2.49). HSQC correlation signals were assigned by comparing with the literature [37, 38].

The molar mass distribution of the xylans was determined by size exclusion chromatography in 50 mM phosphate buffer, pH 8.0 eluted at 0.3 mL/min, 30 °C, using a Biogel P30 column (150 mm × 15 mm I.D.). Samples were dissolved in water, and the concentration was adjusted to give the same absorbance for all samples after the phenol–sulfuric acid reaction (absorbance = 2.0). A total of 400 μL of sample was applied to the column, and eluted fractions were assayed using a phenol–sulfuric acid reagent to determine total sugars [39]. The molar mass markers used in the column system consisted of glucose, cobalamin, dextran T6 (Pharmacosmos), and protein standards (lysozyme, carbonic anhydrase from Sigma).

The solubility of xylans was tested by mixing 5% (w/v) of the solid fractions with water under 120 rpm agitation at 60 °C for 6 h. Prepared suspensions were centrifuged for 20 min at 23,600×*g*, and the xylan content in the supernatant was determined by converting it into the monomeric sugars by acid hydrolysis [40] followed by monomer determination by HPLC, as previously described. Total concentrations of xylose were recorded and used to calculate the xylan solubility in water.

### Saccharification of residual solids with commercial cellulases

Glucan and xylan hydrolysis of alkaline-sulfite-pretreated bagasse, before and after xylan extraction, was assayed at 5% consistency in 50 mM sodium acetate buffer at pH 4.8 (with sodium azide 0.01%) at 45 °C under agitation at 120 rpm. Commercial cellulases at enzyme loads of 2.5 FPU/g of glucan and 5 IU β-glucosidase/g of glucan were used. Hydrolysates were sampled along hydrolysis over 72 h and were analyzed for glucose and xylose contents by HPLC as previously described. Data were reported as glucan and xylan conversion levels based on the monomers released to the solution and the original amounts of glucan and xylan from each sample.

## Results and discussion

### Alkaline-sulfite pretreatment and xylan extraction

Sugarcane bagasse was pretreated in a biorefinery concept designed to recover hemicellulose in polymeric form before enzymatic hydrolysis of the glucan component. Pretreatment was set as an alkaline-sulfite chemithermomechanical delignification process previously described to retain significant hemicellulose portions [5, 8]. The originally described pretreatment [5] was modified to avoid washing of the pretreated material to maximize hemicellulose in the unwashed solids instead of releasing it to the processed wastewaters. The chemical composition of the pretreated material (Table 1) and mass balance for the bagasse components (Additional file 1: Figures S1–S5) revealed that the pretreatment removed 43% of lignin and 8% of xylan from the original bagasse, leaving unwashed solids enriched in glucan and xylan. Xylan removal during pretreatment was lower than usually reported for this process (25–30%) [5], confirming that unwashed solids retained more hemicelluloses. This result is relevant to avoid losses of biomass components in the processed wastewater (Additional file 1: Figures S1–S5). In cases in which lignosulfonates are recovered from the liquid fraction [10], non-washing of the pretreated material also provides less xylan contamination in the final product.

**Table 1:** Chemical composition of sugarcane bagasse before and after alkaline-sulfite pretreatment, and glucurono-arabinoxylans (GAX) extracted from the pretreated material

Components (g/100 g of sample on dry basis)	Untreated bagasse	Unwashed pretreated bagasse	Alkaline-extracted GAX (Hoije method)	Alkaline-extracted GAX (Lopez method)	Enzymatically extracted GAX
Total lignin	21.1 ± 0.4	13.6 ± 0.2	8.4 ± 0.1	10.2 ± 0.2	< 4
Glucan	40.4 ± 0.2	45.2 ± 0.3	5.23 ± 0.1	4.4 ± 0.1	2.2 ± 0.1
Xylan	22.4 ± 0.3	23.2 ± 0.3	61.7 ± 0.8	64.2 ± 0.2	72.5 ± 0.1
Arabinosyl groups	2.6 ± 0.1	2.8 ± 0.1	7.7 ± 0.1	6.8 ± 0.1	11.5 ± 0.8
Uronic acid groups	2.5 ± 0.2	2.4 ± 0.4	6.5 ± 0.1	5.7 ± 0.1	13.3 ± 0.8
Acetyl groups	3.3 ± 0.9	0.2 ± 0.0	nd	nd	nd
Extractives	4.5 ± 0.5	5.4 ± 0.1	na	na	na

*na* not analyzed, *nd* not detected

Xylan structures present in sugarcane bagasse are mainly glucurono-arabinoxylans (GAX) [20, 22, 41]. Therefore, GAX contents were calculated as the sum of xylan (Xyl), arabinosyl (Ara), acetyl (Ac), and glucuronic acid (Uro) residues. GAX contents in the pretreated and untreated materials were quite similar, at 29–31%, respectively (Table 1). Xylose was the most abundant sugar of the pretreated solids, representing 23.2%. In addition, arabinosyl (2.8%) and glucuronic acid (2.4%) side groups were important components in pretreated solids. In contrast, acetyl groups were almost completely removed after pretreatment, decreasing from 3.3% in the untreated bagasse to 0.2% in the pretreated material.

The unwashed pretreated bagasse was used for GAX extraction via alkaline and enzymatic methods. The alkaline methods (Lopez and Hoije procedures) provide high hemicellulose recovery yield, preserving the hemicellulose polymeric form [23]. The enzymatic method was attempted to provide a final product with a higher purity [34].

Table 2 shows GAX crude yields, mass balance for GAX components, and GAX recovery percentages. GAX recovery yield increased following the order: enzymatic (22%), Hoije (45%), and Lopez (53%) extraction procedures. Chimphango et al. [23] evaluated GAX extraction from untreated sugarcane bagasse by alkaline methods, reporting a recovery yield of 47% for Hoije method and almost half for the Lopez method. Here, the highest GAX recovery (53%) was observed when alkaline-sulfite-pretreated bagasse was extracted by the Lopez method (Table 2). Data suggest that more GAX was dissolved by increasing the extraction temperature (Lopez method). Otherwise, the amount of extracted GAX did not increase as a function of the chlorite pre-delignification procedure employed in the Hoije method, likely because the alkaline-sulfite-pretreated bagasse was already partially delignified during pretreatment (Table 1). The unwashed pretreated material could also have retained lignosulfonates acting as surfactants, increasing the diffusion of alkali into the fibers and consequently increasing the solubilization of GAX in the Lopez extraction method [42, 43].

**Table 2:** Glucurono-arabinoxylans (GAX) crude yields, mass balance for GAX components, and recovery percentages from alkaline-sulfite-pretreated sugarcane bagasse

Recovered material or component (g/100 g of original bagasse)	Untreated bagasse	Pretreated bagasse	Alkaline-extracted GAX (Hoije method)	Alkaline-extracted GAX (Lopez method)	Enzymatically extracted GAX
Crude mass	100	88.6	18.1	21.4	7.0
Xylan	22.4	20.6	11.2	13.7	5.1
Arabinosyl groups	2.6	2.5	1.4	1.5	0.8
Uronic acid groups	2.5	2.1	1.2	1.2	0.9
Acetyl groups	3.3	0.2	0.0	0.0	0.0
Total GAX	30.8	25.4	13.8	16.4	6.8
GAX recovery (%)	100.0	82.3	44.6	53.3	22.1

Extraction of GAX assisted by endo-xylanase resulted in low yield (22%), likely due to mild reaction conditions (pH 8) and the low xylanase accessibility to the xylan backbone, even after sugarcane bagasse pretreatment. The lignin in the pretreated bagasse can also decrease the xylanase action owing to non-specific adsorption of the enzyme on the lignin surface and to enzyme inhibition by soluble products generated during the pretreatment, such as sugars and phenolic compounds [44, 45]. Despite low extraction yield, the enzymatic extraction method provided the GAX with the lowest contamination with lignin and glucan (Tables 1, 2). For example, GAX extracted using alkaline methods contained significant residual lignin (8–10%) and glucan (4–5%), whereas enzymatically extracted GAX contained less than 4% lignin and only 2.2% glucan. The color appearances of the prepared GAXs were also substantially different among samples, with enzymatically extracted GAX as a white powder contrasting with dark-brown powders of alkaline-extracted GAXs (Table 1).

### Characterization of isolated xylans

The chemical compositions of the extracted GAXs (Table 1) permitted estimations of the molar ratios of side groups in the GAX backbone. The arabinose/xylose ratio in GAX contained in pretreated sugar cane bagasse (0.12) was almost the same in alkaline-extracted GAXs (0.12 and 0.11 in the Hoije and Lopez methods, respectively). The uronic acid/xylose ratios diminished in the alkaline-extracted GAXs (0.09, 0.08, and 0.06 in pretreated bagasse, and GAXs from Hoije and Lopez methods, respectively), suggesting that the high NaOH load (40%, m/m) in the extraction procedure removed appendage groups from GAX (especially uronic acids). In contrast, the enzymatically extracted GAX presented increased arabinose/xylose and uronic acid/xylose ratios (0.16 and 0.13, respectively), likely because the procedure employed a low alkaline charge, and the commercial xylanase lacks accessory enzymes able to remove appendage groups from the xylan backbone [46].

**Fig. 1:**
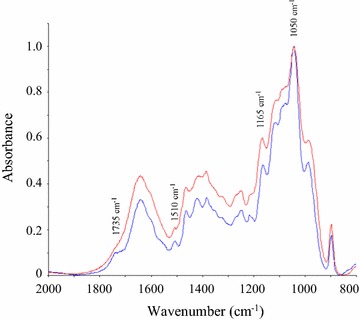
FTIR spectra of alkali-extracted GAXs. Blue and red lines refer to alkali-extracted GAXs by Lopez and Hoije methods, respectively. Enzymatically extracted GAX was not analyzed because it contained small amounts of residual phosphate buffer, which interfered in the FTIR bands of this sample

FTIR spectra of the alkaline-extracted GAXs are shown in Fig. 1. Band assignments were based on previous hemicellulose investigations [21, 47, 48], showing strong signals at 1050 and 1165 cm^−1^, characteristic of C–O and C–C stretching from carbohydrates. The weak band at 1510 cm^−1^ was attributed to lignin contamination, which corroborates the chemical compositions shown in Table 1. The shoulder at 1735 cm^−1^ was attributed to carbonyl/carboxyl stretching, corroborating the occurrence of glucuronic acid in evaluated GAXs. Feruloyl and acetyl esters attached to the xylan backbone of sugarcane samples [20] could also absorb in the 1735 cm^−1^ region, but the alkaline conditions used in the pretreatment and GAX preparation procedures suggest that these esters did not resist the processing conditions, as indicated by the absence of acetyl groups in the evaluated GAXs (Table 1).

**Fig. 2:**
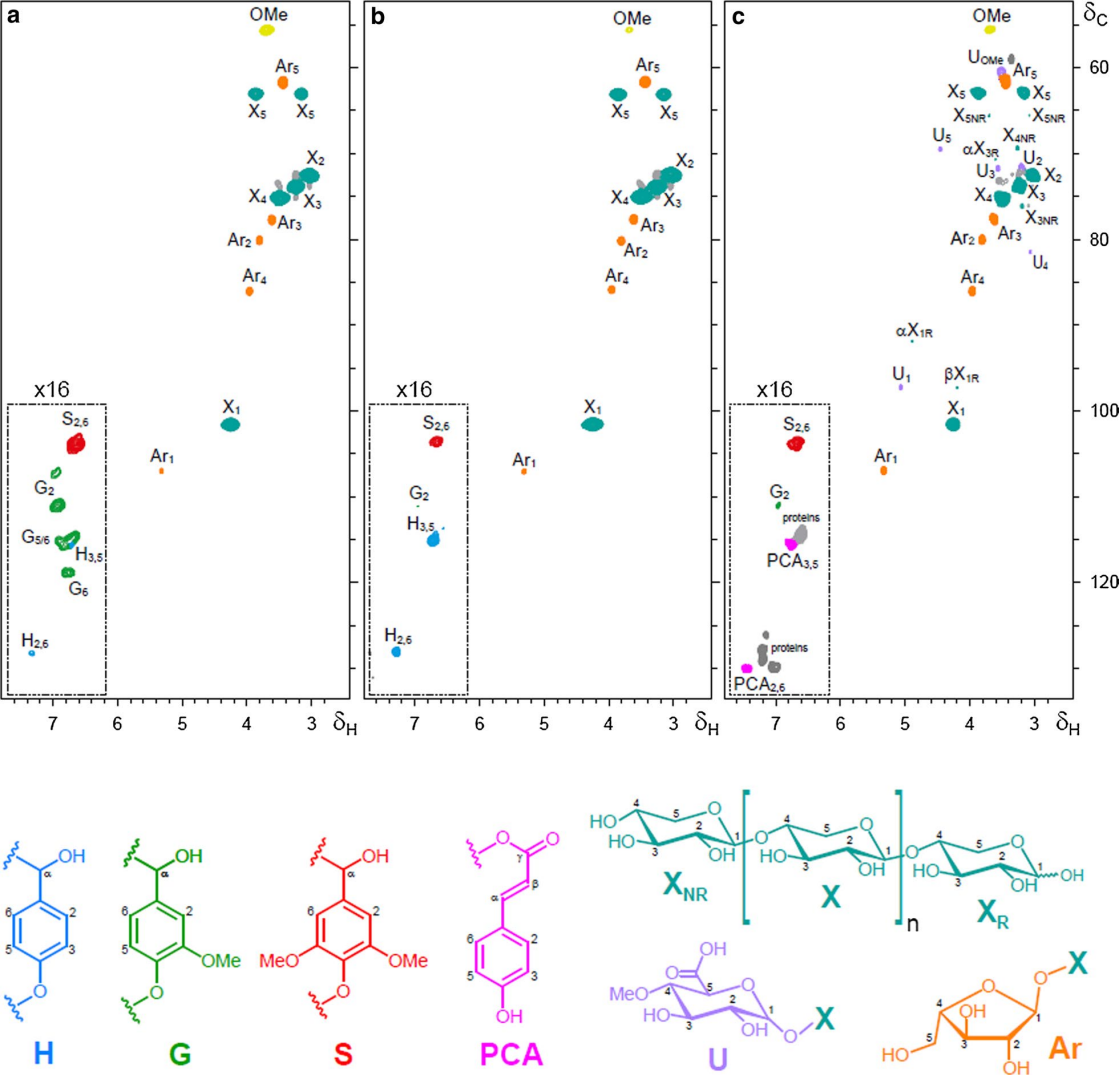
2D-NMR-HSQC spectra (*δ*_C_/*δ*_H_ 52–135/2.4–7.8) of the GAX samples extracted from pretreated sugarcane bagasse. **a** GAX extracted by Hoije method; **b** GAX extracted by Lopez method; **c** GAX extracted by enzymatic treatment. The inset shows a 16-fold amplification of the aromatic region with the main lignin aromatic signals. The main carbohydrate and lignin structures identified are **X**, xylose units; **Ar**, arabinose units; **U**, glucuronic acid units; **H**, *p*-hydroxyphenyl units; **G**, guaiacyl units; **S**, syringyl units; **PCA**, *p*-coumaric acid. See Additional file 2: Table S1 for signal assignments

Prepared samples were also analyzed by 2D-HSQC-NMR, as shown in Fig. 2. The main carbohydrate and lignin correlation signals assigned in the HSQC spectra are listed in Additional file 2: Table S1, and the main substructures found are depicted in Fig. 2. The HSQC spectra of the GAX samples showed strong signals from carbohydrates, whereas lignin signals were barely detected and could only be observed after the amplification of the aromatic region of the spectra. The most important carbohydrate signals in the spectra corresponded to the C_1_/H_1_, C_2_/H_2_, C_3_/H_3_, C_4_/H_4_, and the double C_5_/H_5_ correlations of xylans (**X**_**1**_, **X**_**2**_, **X**_**3**_, **X**_**4**_, **X**_**5**_). Signals from *O*-acetylated xylans, which were present in important amounts in the spectra of the starting sugarcane sample (data not shown), were absent from the spectra of the isolated xylans, indicating that acetate groups attached to xylans have been completely hydrolyzed and removed during the pretreatment and/or extraction processes. Signals for the C_1_/H_1_ C_2_/H_2_, C_3_/H_3_, C_4_/H_4_, and C_5_/H_5_ correlations of arabinosyl side groups (**Ar**_**1**_, **Ar**_**2**_, **Ar**_**3**_, **Ar**_**4**_, **Ar**_**5**_) were also observed together with the xylan signals in the three GAX samples. The arabinose/xylose ratios of the alkaline-extracted GAXs were approximately 0.07, whereas the arabinose/xylose ratio of the enzymatic extracted GAX was higher, approximately 0.16 in agreement with the data calculated from wet chemical characterization of the sample (Table 1). These data may indicate a partial removal of xylose units during the enzymatic treatment with xylanases or preferential dissolution of more substituted fractions of the original xylan. The spectrum of the enzymatic extracted GAX (Fig. 2c) also revealed the occurrence of other carbohydrate signals, including the correlation signals for 4-*O*-methyl-α-d-glucuronic acid (**U**_**1**_, **U**_**2**_, **U**_**3**_, **U**_**4**_, **U**_**5**_), which were absent from the spectra of the alkaline-extracted xylans obtained using the Hoije and Lopez methods. Uronic acids and arabinosyl groups were detected at similar concentrations by wet chemical characterization. These observations suggest that the carbazole method employed for uronic acids determination tends to overestimate this component in the analyzed xylan. In addition, the presence of signals for the reducing (**X**_**R**_) and non-reducing (**X**_**NR**_) ends of xylans in the enzymatically extracted GAX indicates that this sample contains shorter chains as a consequence of partial depolymerization during the isolation process by the use of xylanases.

Signals for residual lignin could also be observed in the HSQC spectra of the three GAX samples but only after the amplification (16×) of the signal (see insets of Fig. 2). The main cross-signals in the aromatic regions of the HSQC spectra corresponded to the different lignin units and to the *p*-coumaric (**PCA**) acid. Signals from guaiacyl (**G**) and syringyl (**S**) units, together with lower amounts of signals for *p*-hydroxyphenyl (**H**) lignin units, were observed in the spectra of the GAX isolated using the Hoije method, with an S:G:H composition of 44:50:6 and an S/G ratio of ~ 0.9 (Fig. 2a). However, mostly signals from H- and S-lignin units could be observed in the GAX isolated using the Lopes method, with only traces of signals for G-lignin, with an S:G:H composition of 54:15:31 and an S/G ratio of 3.6 (Fig. 2b). Interestingly, the GAX isolated by enzymatic treatment presented signals from S- and G-lignin units (S/G ratio of ~ 1.9), together with some signals from residual **PCA**, suggesting PCA retention owing to the mild extraction procedure. Significant amounts of signals from proteins, most likely arising from the xylanases used in the isolation process, could also be clearly distinguished in the spectrum of the enzymatically extracted GAX (Fig. 2c).

**Fig. 3:**
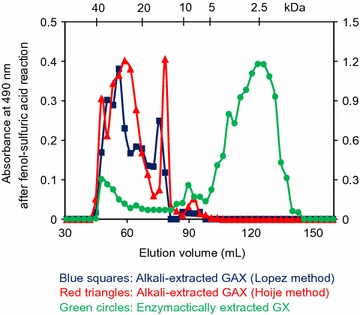
Size exclusion chromatography of glucurono-arabinoxylans (GAX) extracted from alkaline-sulfite-pretreated sugarcane bagasse. Left absorbance scale refers to alkaline-extracted GAXs. Right absorbance scale refers to enzymatically extracted GAX

The molar mass distribution of the extracted GAXs was evaluated by size exclusion chromatography (Fig. 3). The chromatograms show heterogeneous distribution of several GAX polymers. Alkaline-extracted GAXs presented significant fractions with molar masses higher than 12 kDa. In contrast, enzymatically extracted GAX presented a wide chromatogram peak, with molar masses lower than 6 kDa, in agreement with 2D-NMR data suggesting the presence of shorter xylan chains. In this case, the small fraction with high molar mass (eluting from 45 to 60 mL) corresponded to only 28% of the chromatogram area.

The solubility in water of the isolated GAXs was determined and compared with that of beechwood xylan, a commercial xylan with a high degree of substitution (9.4% of uronic acids and 6.9% of other sugars) (http://www.megazyme.com). Beechwood xylan presented the highest solubility in water (2.18 g/L), followed by the enzymatically extracted GAX (0.77 g/L) and the alkaline-extracted GAXs (0.52 and 0.28 g/L for the Hoije and Lopez methods, respectively). The highest solubility in water of the sugarcane bagasse GAX corresponded to the polymer with the lowest molar mass and the highest substitution degree as well as the lowest lignin content (enzymatically extracted GAX). Upon comparing the two alkaline-extracted GAXs, which presented similar molar mass distributions and branching, the presence of residual lignin and its structural characteristics seems to determine the differences in solubility in aqueous media [49]. In fact, the less-soluble GAX (obtained using the Lopez method) exhibited the highest residual lignin content (Table 1), and a lignin structure including significant amounts of more hydrophobic H units (Fig. 2b).

### Enzymatic conversion of residual glucan

**Fig. 4:**
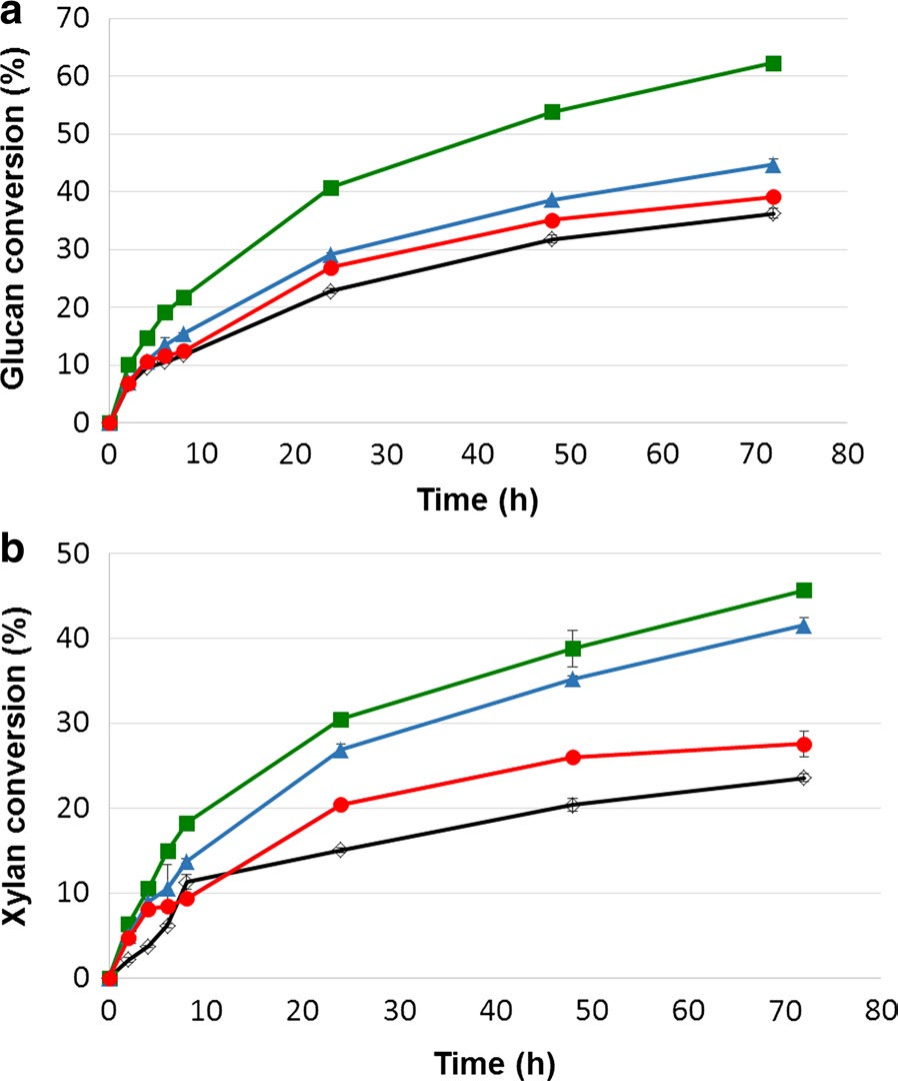
Enzymatic hydrolysis of solids remaining after sugarcane bagasse pretreatment and GAX extraction by varied procedures. Open symbol represents samples before GAX extraction (diamonds) and closed symbols after GAX extraction by varied methods: (green rectangles) Hoije; (blue triangles) Lopes; and (red circles) enzymatic

Pretreated sugarcane bagasse and the solid residues remaining after GAX extraction were saccharified by commercial cellulases. Table 3 presents the chemical compositions of solids remaining after GAX extraction. Sugarcane bagasse pretreatment and GAX extraction resulted in residual solids significantly enriched in glucan. Therefore, a low cellulase dosage (2.5 FPU/g glucan in the substrate) was used to differentiate the performance of each sample under enzymatic hydrolysis (Fig. 4).

**Table 3:** Chemical composition of residual solids after sugarcane bagasse pretreatment and GAX extraction by varied methods

Residual solids after GAX extraction	Crude yield (%)	Lignin (%)	Glucan (%)	Xylan (%)	Arabinosyl groups (%)
Hoije method	43.7	7.2 ± 0.7	74.2 ± 0.4	14.7 ± 0.2	0.5 ± 0.1
Lopez method	45.2	10.5 ± 0.4	78.9 ± 0.4	9.2 ± 0.2	0.3 ± 0.0
Enzymatic method	67.9	15.4 ± 0.2	50.8 ± 0.6	22.1 ± 0.2	2.3 ± 0.0

The glucan component from the residual solids after GAX extraction using the Hoije method was promptly converted to glucose, even with a low cellulase dosage (70% glucan conversion after 72 h), while for the other samples, a maximum of 40% glucan conversion was attained (Fig. 4a). The varied performance of the solid residue from the Hoije method can be associated with the pre-delignification step applied over the pretreated bagasse. This process decreased the lignin content in the residual solids to 7%, effectively increasing the accessibility of the enzymes to the cellulose microfibrils. In contrast, the solid residues obtained after GAX extraction by the Lopez and the enzymatic methods presented higher residual lignin contents (10 and 15%, respectively).

Figure 4b indicates that xylans remaining in the residual solids were still converted to xylose by the cellulase cocktail, which presents high xylanase activities [44, 50, 51]. Xylan conversion to xylose from the solid residues resulting from GAX extraction was low (40 and 25% for alkaline and enzymatically extracted residues, respectively), probably associated with the low enzyme dosage used in the saccharification experiments.

### Mass balance for the sugarcane bagasse components

New biorefinery concepts are necessary to drive industrial use of lignocellulose biomass components. Xylan recovery before enzymatic hydrolysis of the glucan component is a way to add value to the hemicellulose fraction [19, 52]. This biorefinery design also avoids changes to the currently established hexose fermentation processes since final hydrolysates produced from sugarcane bagasse will contain mainly glucose instead of a mixture of glucose and xylose [13]. Mass balance for bagasse components permits an overall view of each component fate during processing (Additional file 1: Figures S1–S5).

**Fig. 5:**
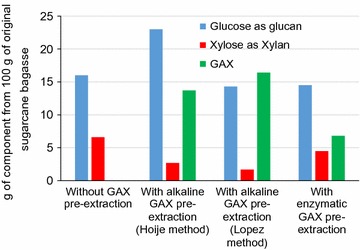
Mass balance for solid fractions, wastewaters, and the final monosaccharide hydrolysate during sugarcane bagasse processing by a biorefinery concept including GAX extraction before enzymatic hydrolysis of the polysaccharides

Conventional processing of sugarcane bagasse using alkaline-sulfite chemithermomechanical pretreatment provides a suitable substrate for direct enzymatic hydrolysis. The process provides up to 34 g of glucose and 14 g of xylose (both expressed in their polymeric forms) from 100 g of original sugarcane bagasse. This result is obtained with extensively washed pretreated material and high enzyme dosage during enzymatic hydrolysis (16 FPU/g of glucan) [5]. Using unwashed substrate and reducing the enzyme dosage to 2.5 FPU/g of glucan during hydrolysis, the glucose and xylose yields were 13 and 5 g per 100 g of original bagasse, respectively (Additional file 1: Figure S5). Under the current biorefinery described scenarios, GAX extraction from unwashed pretreated material was attempted to add value to the process products while maintaining low enzyme dosages in the final hydrolysis step (2.5 FPU/g of glucan in the substrate). The main task was to generate GAX as a co-product in the process and simultaneously decreasing xylose in the enzymatic hydrolysate. Figure 5 summarizes the amounts of GAX, glucose, and xylose recovered from sugarcane bagasse processed in the four different scenarios currently evaluated. In all cases, avoiding washing after pretreatment preserved carbohydrates in the solid fraction with a corresponding decreased carbohydrate loss in the first processed wastewater (Additional file 1: Figures S1–S3). GAX extraction from the unwashed pretreated material resulted in varied GAX yields depending on the extraction procedure. After GAX extraction via alkaline procedures, the solid residues treated with low cellulase dosages yielded similar or even higher glucose levels than those obtained from direct conversion of the unwashed pretreated material (Fig. 5). Enzymatic GAX extraction provided a high-purity GAX at low yield, but the glucose levels in the final hydrolysates were still similar to those obtained from direct hydrolysis of the unwashed pretreated material. The final hydrolysates from the solid residues obtained after alkaline GAX extraction methods presented high glucose/xylose ratios (8.4–8.5) (Additional file 1: Figures S2, S3), whereas the residual solids after enzymatic GAX extraction and the unwashed pretreated material presented low glucose/xylose ratios (2.4—Additional file 1: Figure S4, 3.2—Additional file 1: Figure S1, respectively).

## Conclusions

Over 90% of the xylan was preserved in the sugarcane bagasse after chemithermomechanical pretreatment avoiding excessive washing of the pretreated solids. Residual xylans were extracted by three processes with different yields and purity levels. The xylans isolated using alkaline methods used high alkali charge and produced high-molecular-weight xylans, with high Ara/Xyl ratios and with 8–10% residual lignin. In contrast, the enzymatic extraction process took a light-colored and low-molecular-weight product, almost free of residual lignin, and Ara/Xyl and Uro/Xyl ratios higher than alkaline xylans. While enzymatic extraction had the advantage of producing high-purity xylans, the yield and degree of polymerization were relatively low. This product could be a highly attractive material for deposition onto cellulosic pulps for increasing their xylan content, thereby providing added value to xylan-bagasse. The overall mass balance of the process shows that by removing xylan in high yield (alkaline methods), the glucan-enriched solid residue can be readily hydrolyzed with low cellulase loads, generating hydrolysates with a high glucose/xylose ratio, suggesting the possibility of direct use in conventional hexose fermentation processes.

## Additional files

**Additional file 1: Figure S1.** Mass balance for sugarcane bagasse components during alkaline-sulfite chemothermomechanical pretreatment followed by GAX extraction based on the enzymatic method and enzymatic hydrolysis of unwashed pretreated solids. **Figure S2.** Mass balance for sugarcane bagasse components during alkaline-sulfite chemothermomechanical pretreatment followed by GAX extraction based on the De Lopez method and enzymatic hydrolysis of unwashed pretreated solids. **Figure S3.** Mass balance for sugarcane bagasse components during alkaline-sulfite chemothermomechanical pretreatment followed by GAX extraction based on the Hoijemethod and enzymatic hydrolysis of unwashed pretreated solids. **Figure S4.** Mass balance for sugarcane bagasse components during alkaline-sulfite chemothermomechanical pretreatment followed by enzymatic hydrolysis of unwashed pretreated solids. **Figure S5.** Mass balance for sugarcane bagasse components during alkaline-sulfite chemothermomechanical pretreatment followed by extensive washing and enzymatic hydrolysis of pretreated solids.

**Additional file 2: Table S1.** Assignment of the carbohydrate and lignin ^13^C/^1^H correlation signals in the 2D HSQC NMR spectra of the GAX samples.
