# Supplementary material for: Xylan extraction from pretreated sugarcane bagasse using alkaline and enzymatic approaches
Source: Biotechnol Biofuels. 2017 Dec 7;10:296. doi: 10.1186/s13068-017-0981-z (PMC5719793; doi:10.1186/s13068-017-0981-z)
Supplement: Supplementary file 1 — Additional file 1: Figure S1. Mass balance for sugarcane bagasse components during alkaline-sulfite chemothermomechanical pretreatment followed by GAX extraction based on the enzymatic method and enzymatic hydrolysis of unwashed pretreated solids. Figure S2. Mass balance for sugarcane bagasse components during alkaline-sulfite chemothermomechanical pretreatment followed by GAX extraction based on the De Lopez method and enzymatic hydrolysis of unwashed pretreated solids. Figure S3. Mass balance for sugarcane bagasse components during alkaline-sulfite chemothermomechanical pretreatment followed by GAX extraction based on the Hoijemethod and enzymatic hydrolysis of unwashed pretreated solids. Figure S4. Mass balance for sugarcane bagasse components during alkaline-sulfite chemothermomechanical pretreatment followed by enzymatic hydrolysis of unwashed pretreated solids. Figure S5. Mass balance for sugarcane bagasse components during alkaline-sulfite chemothermomechanical pretreatment followed by extensive washing and enzymatic hydrolysis of pretreated solids. [file 13068_2017_981_MOESM1_ESM.pdf]

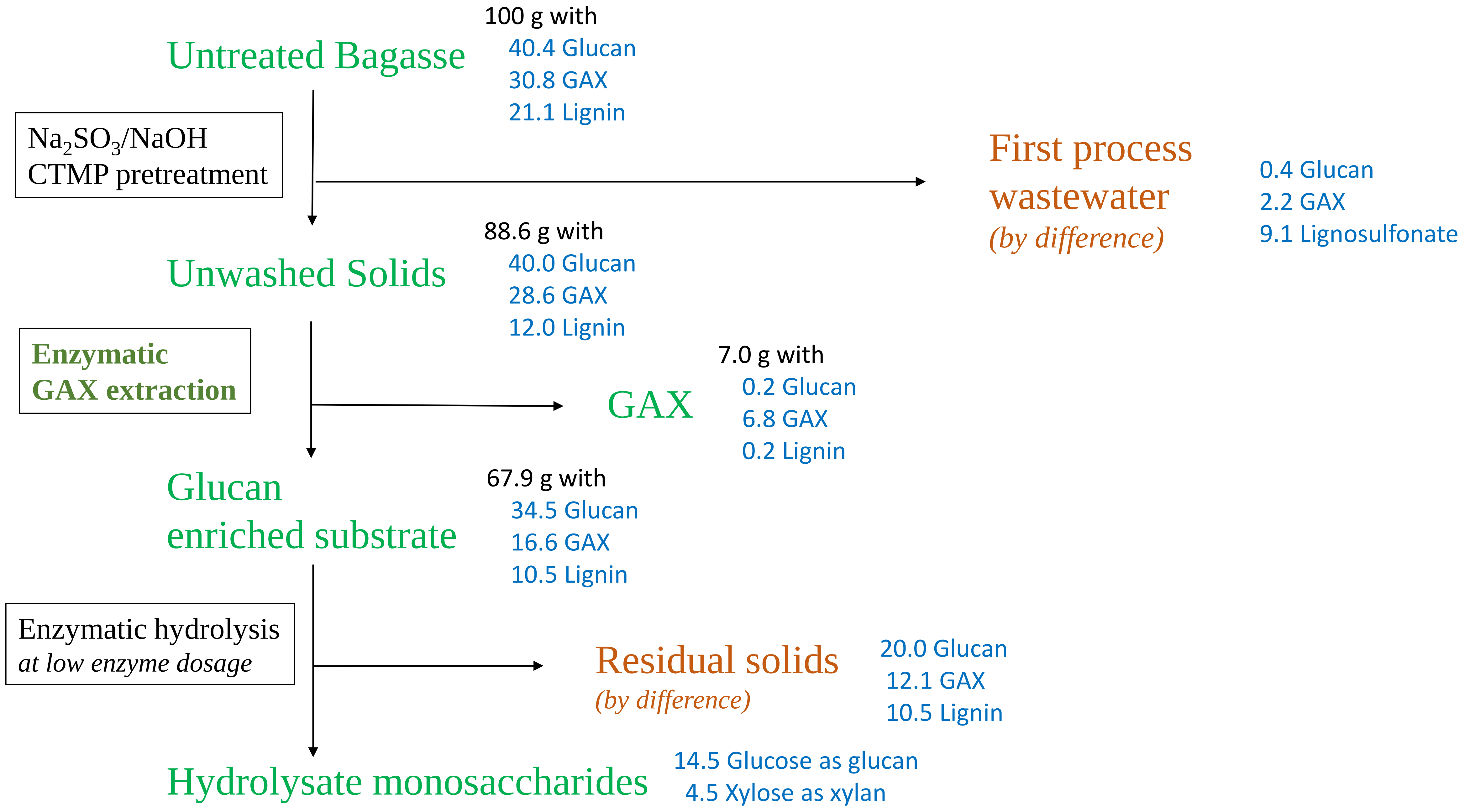

Figure S1. Mass balance for sugarcane bagasse components during alkaline-sulfite chemothermomechanical pretreatment followed by GAX extraction based on the enzymatic method and enzymatic hydrolysis of unwashed pretreated solids

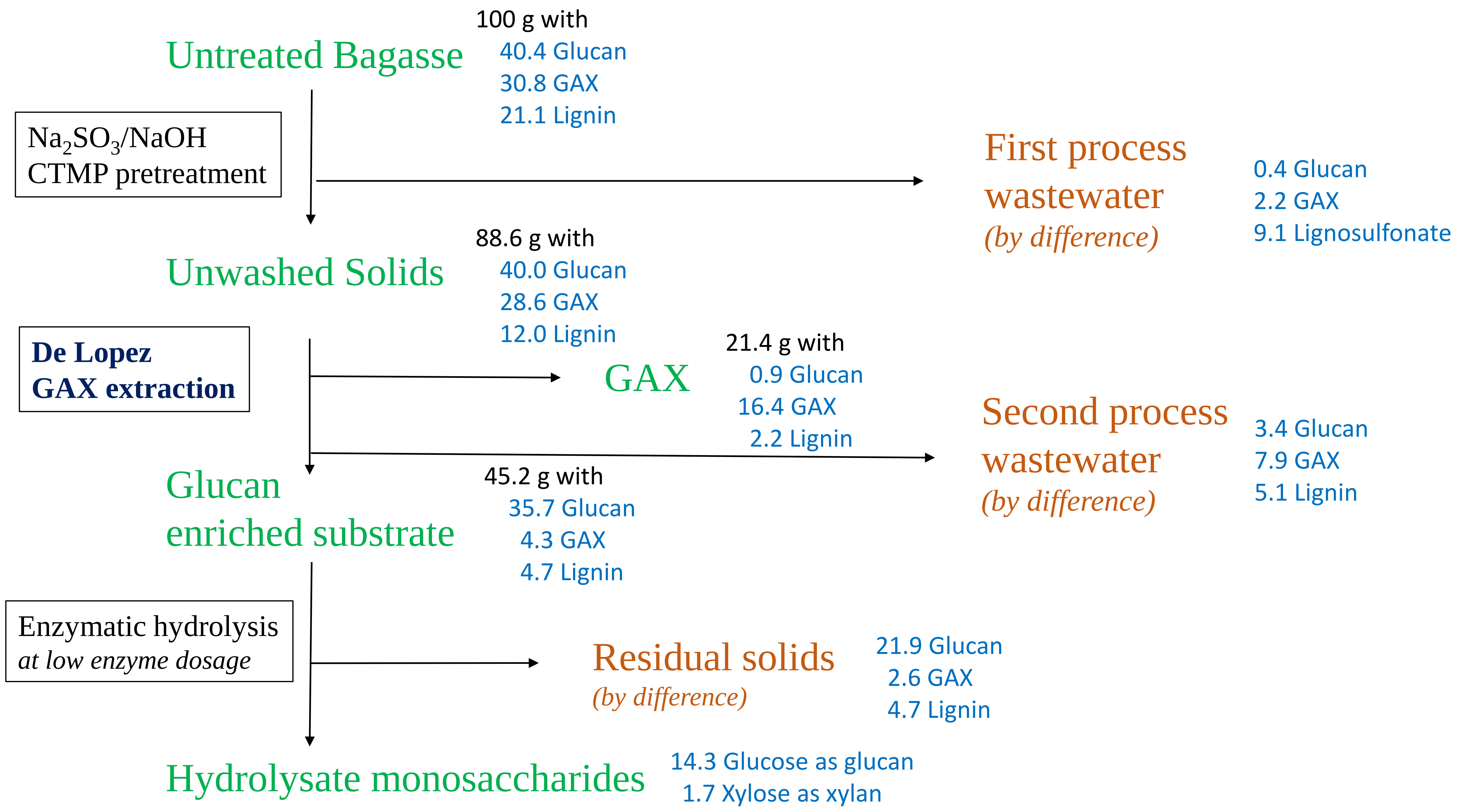

Figure S2. Mass balance for sugarcane bagasse components during alkaline-sulfite chemothermomechanical pretreatment followed by GAX extraction based on the De Lopez method and enzymatic hydrolysis of unwashed pretreated solids

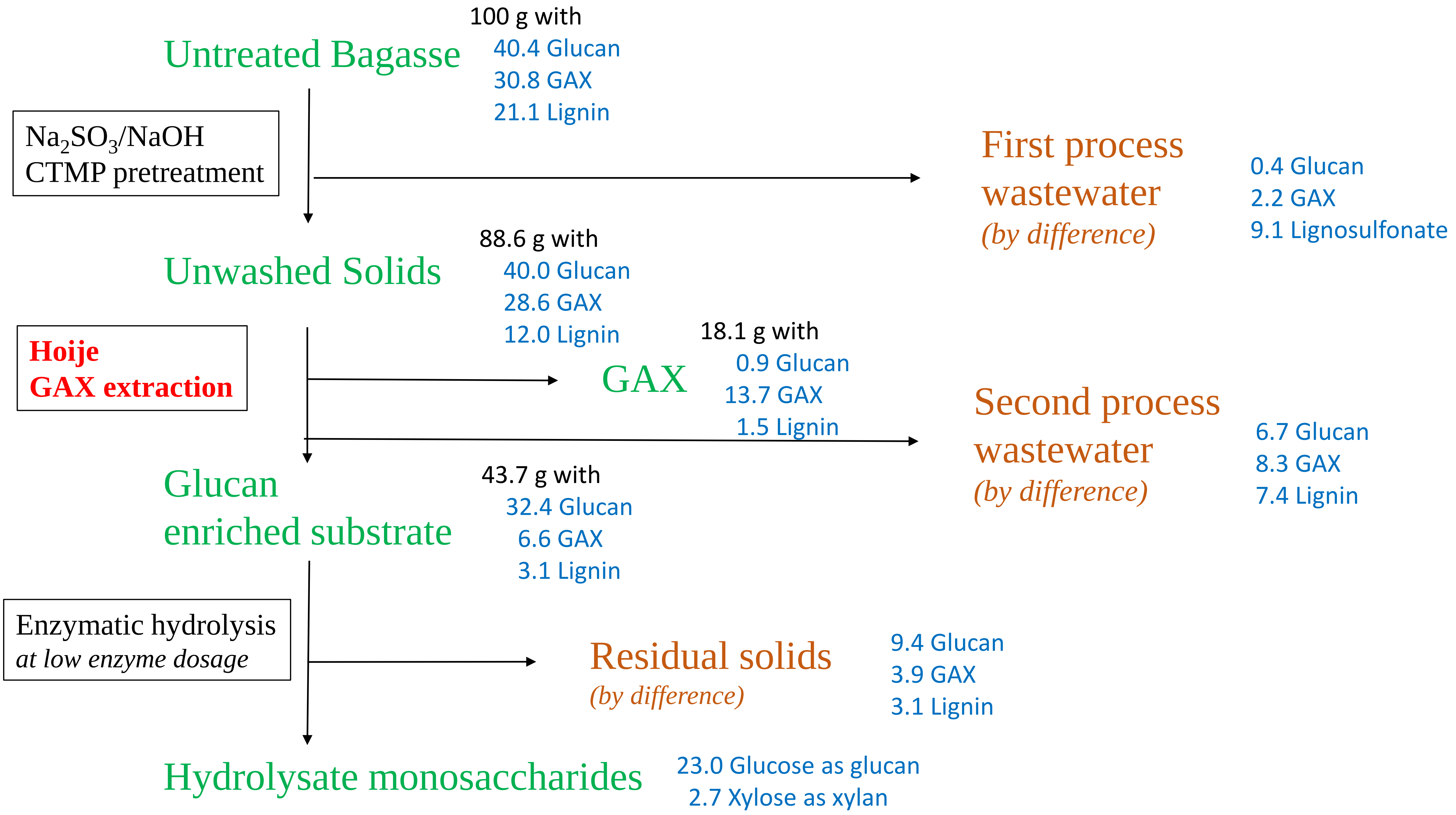

Figure S3. Mass balance for sugarcane bagasse components during alkaline-sulfite chemothermomechanical pretreatment followed by GAX extraction based on the Hoije method and enzymatic hydrolysis of unwashed pretreated solids

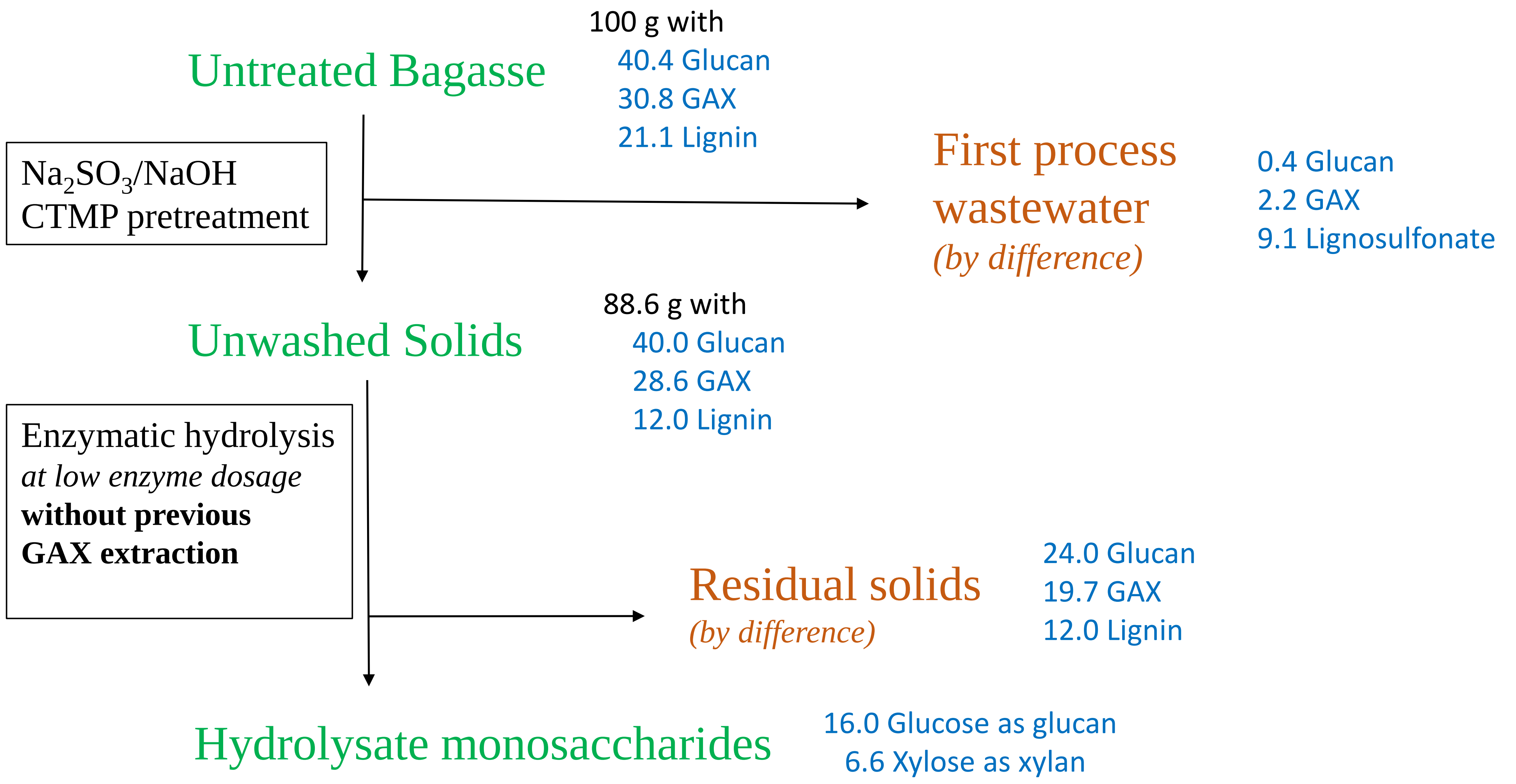

Figure S4. Mass balance for sugarcane bagasse components during alkaline-sulfite chemothermomechanical pretreatment followed by enzymatic hydrolysis of unwashed pretreated solids

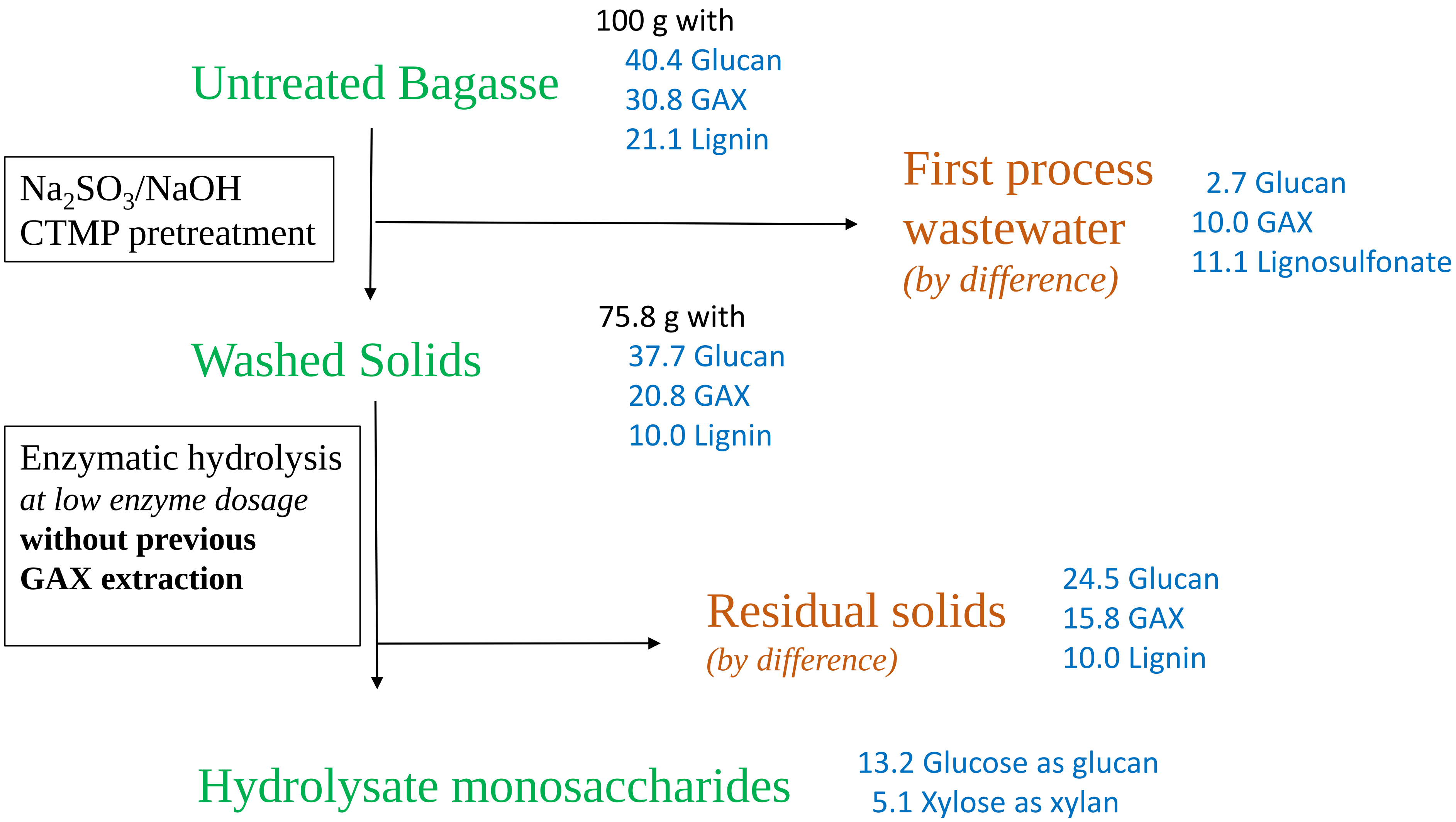

Figure S5. Mass balance for sugarcane bagasse components during alkaline-sulfite chemothermomechanical pretreatment followed by extensive washing and enzymatic hydrolysis of pretreated solids
