# Supplementary material for: Xylan extraction from pretreated sugarcane bagasse using alkaline and enzymatic approaches
Source: Biotechnol Biofuels. 2017 Dec 7;10:296. doi: 10.1186/s13068-017-0981-z (PMC5719793; doi:10.1186/s13068-017-0981-z)
Supplement: Supplementary file 2 — Additional file 2: Table S1. Assignment of the carbohydrate and lignin 13C/1H correlation signals in the 2D HSQC NMR spectra of the GAX samples. [file 13068_2017_981_MOESM2_ESM.pdf]

Table S1. Assignment of the carbohydrate and lignin $^{13}C/^{1}H$ correlation signals in the 2D HSQC NMR spectra of the GAX samples.

Label	δ_C/δ_H	Assignment
Carbohydrate signals		
U_{OMe}	60.4/3.51	C/H of methoxyls in 4-*O*-methylglucuronic acid
Ar_5	61.6/3.44	C_5/H_5 in α-L-arabinofuranoside
X_5	62.8/3.20 and 3.86	C_5/H_5 in β-D-xylopyranoside
X_{5NR}	65.5/370 and 3.06	C_5/H_5 in β-D-xylopyranoside (NR)
X_{4NR}	69.4/3.27	C_4/H_4 in β-D-xylopyranoside (NR)
U_5	69.5/4.45	C_5/H_5 in 4-*O*-methylglucuronic acid
αX_{3R}	70.2/3.64	C_3/H_3 in α-D-xylopyranoside (R)
U_2	71.8/3.20	C_2/H_2 in 4-*O*-methylglucuronic acid
X_2	72.6/3.02	C_2/H_2 in β- D-xylopyranoside
U_3	73.2/3.55	C_3/H_3 in 4-*O*-methylglucuronic acid
X_3	73.7/3.23	C_3/H_3 in β- D-xylopyranoside
X_4	75.3/3.52	C_4/H_4 in β- D-xylopyranoside
X_{3NR}	76.2/3.20	C_3/H_3 in β-D-xylopyranoside (NR)
Ar_3	77.7/3.62	C_3/H_3 in α-L-arabinofuranoside
Ar_2	80.0/3.80	C_2/H_2 in α-L-arabinofuranoside
U_4	81.4/3.06	C_4/H_4 in 4-*O*-methylglucuronic acid
Ar_4	86.0/3.96	C_4/H_4 in α-L-arabinofuranoside
αX_{1R}	92.1/4.85	C_1/H_1 in α-D-xylopyranoside (R)
U_1	97.2/5.06	C_1/H_1 in 4-*O*-methylglucuronic acid
βX_{1R}	97.3/4.22	C_1/H_1 in β-D-xylopyranoside (R)
X_1	101.6/4.29	C_1/H_1 in β- D-xylopyranoside
Ar_1	107.0/5.32	C_1/H_1 in α-L-arabinofuranoside
Lignin signals		
$-OCH_3$	55.6/3.73	C/H of methoxyls in lignin
$S_{2,6}$	103.8/6.69	C_2/H_2 and C_6/H_6 in etherified syringyl units (S)
G_2	110.9/7.00	C_2/H_2 in guaiacyl units (G)
$H_{3,5}$	114.5/6.62	C_3/H_3 and C_5/H_5 in *p*-hydroxyphenyl units (H)
G_5/G_6	114.9/6.72 and 6.94	C_5/H_5 and C_6/H_6 in guaiacyl units (G)
$PCA_{3,5}$	115.5/6.77	C_3/H_3 and C_5/H_5 in *p*-coumarates (PCA)
G_6	118.7/6.77	C_6/H_6 in guaiacyl units (G)
$H_{2,6}$	128.0/7.23	C_2/H_2 and C_6/H_6 in *p*-hydroxyphenyl units (H)
$PCA_{2,6}$	130.0/7.46	C_2/H_2 and C_6/H_6 in *p*-coumarates (PCA)

R, reducing end units; NR, non-reducing end units
